# AF1q: A Novel Mediator of Basal and 4-HPR-Induced Apoptosis in Ovarian Cancer Cells

**DOI:** 10.1371/journal.pone.0039968

**Published:** 2012-06-26

**Authors:** Paola Tiberio, Elena Cavadini, Maurizio Callari, Maria Grazia Daidone, Valentina Appierto

**Affiliations:** Department of Experimental Oncology and Molecular Medicine, Fondazione IRCCS “Istituto Nazionale dei Tumori”, Milan, Italy; Istituto di Ricerche Farmacologiche Mario Negri, Italy

## Abstract

**Background:**

Fenretinide (4-HPR) is a synthetic retinoid that exhibits potent antitumor and chemopreventive activities against different malignancies, including ovarian tumors. We previously showed that in ovarian cancer cells, 4-HPR induces apoptosis through a signaling cascade starting from reactive oxygen species (ROS) generation and involving endoplasmic reticulum (ER) stress response, Jun N-terminal Kinase (JNK) activation, and induction of the proapoptotic PLAcental Bone morphogenetic protein (PLAB). Since recent studies have shown that the oncogene ALL1-fused from chromosome 1q (AF1q), a retinoic acid target gene, is implicated in apoptosis induction by several therapeutic agents, we investigated its possible involvement in the apoptosis induced by 4-HPR in ovarian cancer cells.

**Methodology/Principal Findings:**

Protein expression analysis, performed in ovarian cancer cells and extended to other histotypes (breast, neuroblastoma, and cervical), revealed that 4-HPR enhanced AF1q expression in cancer cells sensitive to the retinoid but not in resistant cells. Through gene silencing, AF1q was found functionally involved in 4-HPR-induced apoptosis in A2780, an ovarian cancer cell line highly sensitive to retinoid growth inhibitory and apoptotic effects. Inhibition of the signaling intermediates of the 4-HPR apoptotic cascade showed that AF1q upregulation was depended on prior generation of ROS, induction of ER stress response, JNK activation, and PLAB upmodulation. Finally, we found that direct overexpression of AF1q, in the absence of external stimuli, increased apoptosis in ovarian cancer cell lines.

**Conclusions/Significance:**

The study expands the knowledge of the 4-HPR mechanism of action, which has not yet been completely elucidated, identifying AF1q as a novel mediator of retinoid anticancer activity. In addition, we demonstrate, for the first time, that AF1q plays a role in the onset of basal apoptosis in ovarian cancer cells, thus providing new information about the activity of this protein whose biologic functions are mostly unknown.

## Introduction

Retinoids, a large family of natural and synthetic compounds structurally related to vitamin A (retinol), are essential signaling molecules that regulate a wide variety of biological processes such as embryonic development, differentiation, maintenance of epithelial tissue, reproduction, vision, proliferation and apoptosis [1], [2]. N-(4-hydroxyphenyl)retinamide (4-HPR), also known as fenretinide, is a synthetic vitamin A derivative well tolerated in humans that has emerged as one of the most promising retinoids in the clinic for prevention and treatment of numerous malignancies [3]–[7]. 4-HPR has been shown to possibly reduce the occurrence of ovarian cancer and to determine, in premenopausal women, a significant reduction in the risk of a second breast cancer that persists for at least 15 years [3], [6]. Even though 4-HPR mechanisms of action has been an important focus of research over the last decade, the molecular events mediated by the retinoid appear multifaceted and have not been completely elucidated [8]. Unlike classical retinoids (such as all-trans retinoic acid), that often induce differentiation and/or cytostasis by activating the family of nuclear retinoic acid receptors (RARs), 4-HPR has been shown to elicit different biological effects through both retinoid receptor–dependent and –independent mechanisms [9].

In cultured cells, 4-HPR has been shown to inhibit cell proliferation and to promote apoptosis mediated, at least in part, by the generation of reactive oxygen species (ROS) and consequent oxidative stress [8]–[11]. We previously provided evidence that, in ovarian cancer cells, 4-HPR induced apoptosis through a signaling cascade starting from ROS production and involving endoplasmic reticulum (ER) stress response, Jun N-terminal Kinase (JNK) activation, and induction of the proapoptotic PLAcental Bone morphogenetic protein (PLAB, also known as NAG-1, GDF15, MIC-1, PDF, and PTGFB) (ROS → ER stress → JNK → PLAB) [11], [12].

The ALL1-fused from the chromosome 1q (AF1q, also known as MLLT11) gene, located in chromosome 1, band 21, encodes a small protein of 9 kDa with no well-defined functional domains and no similarity to other known proteins [13]. The mechanisms underlying the regulation of AF1q expression have been investigated at mRNA and protein levels. An increase in AF1q mRNA levels has been shown in retinoic acid (RA)-responsive neuroblastoma cells after exposure to the retinoid, thus identifying AF1q as a RA-target gene [14]. More recently, it has been demonstrated that AF1q mRNA expression is directly regulated by microRNA29b [15], whereas the protein is subjected to ubiquitin-mediated degradation by the proteasome in the centrosomal area [16]. AF1q was originally defined as an oncogenic factor implicated in a translocation t(1;11)(q21;q23) responsible for cases of leukemia [13]. Moreover, a high expression level of the gene was reported to be associated with a poor outcome in pediatric acute myeloid leukemia (AML), adult normal cytogenetic AML, and adult myelodysplastic syndrome [17]–[19]. Although the biological functions of AF1q are largely unknown, a potential oncogenic function of the protein has also been suggested in solid tumors such as breast cancer and thyroid oncocytic and testicular germ cell tumors [20]–[23]. In addition to the proposed oncogenic role of AF1q, it has been reported that the protein may regulate the BAD-mediated apoptotic pathway via NF-kB, thus playing a role in the regulation of apoptosis and in drug resistance [24], [25]. A decrease in AF1q expression in conjunction with reduced doxorubicin sensitivity has been observed in human squamous carcinoma cells, and knockdown of the protein has been shown to decrease apoptotic cell death induced by several therapeutic agents, such as doxorubicin and γ radiation [24], [25].

The present study investigated the involvement of AF1q in 4-HPR-induced apoptosis in ovarian tumor cells. We provide evidence that 4-HPR treatment enhances AF1q expression levels in cancer cells sensitive to the retinoid and that such upregulation is an important event of the 4-HPR signaling cascade that leads to apoptosis through ROS generation, ER stress response, JNK activation, and PLAB upregulation. Finally, by AF1q overexpression, we demonstrated a role of AF1q in the onset of basal apoptosis in ovarian cancer cells.

## Results

### 4-HPR Induces AF1q Upregulation in Ovarian Cancer Cells Sensitive to the Retinoid but not in Resistant Cells

**Figure 1:**
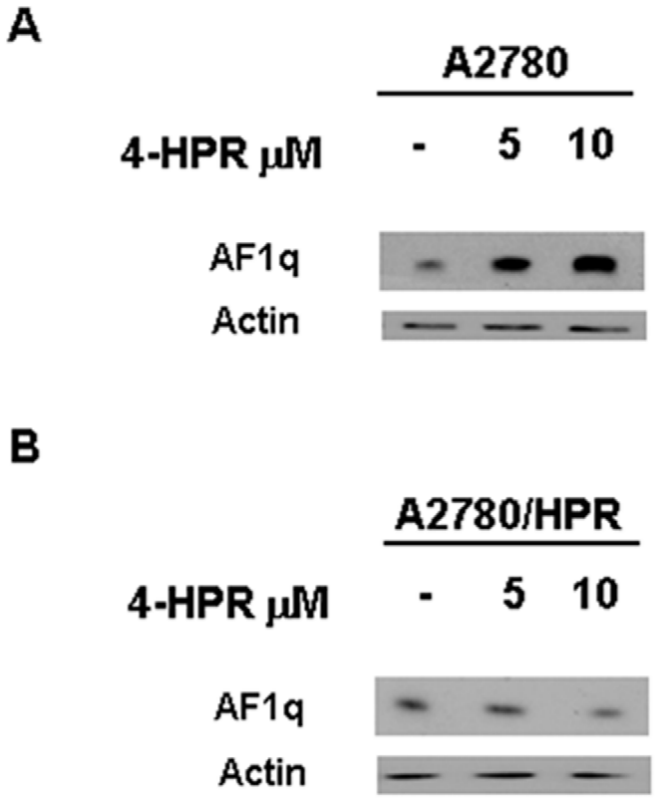
Effects of 4-HPR on AF1q expression in A2780 and A2780/HPR cells. Western blot analysis for AF1q expression in A2780 (A) and A2780/HPR (B) cells treated for 24 hours with 5 and 10 µM 4-HPR. As a control for loading, the blots were incubated with actin antibody.

Since AF1q was shown to play a role in the apoptosis induced by several therapeutic agents and to be regulated by RA [14], [24], [25], we sought to investigate the potential contribution of the protein in the apoptotic activity of the synthetic retinoid 4-HPR. We analyzed the effect of 4-HPR treatment on AF1q expression in the human ovarian cancer cell line A2780, which is highly sensitive to the antiproliferative and apoptotic effects of the retinoid [26], [27], and in its counterpart A2780/HPR, which has induced resistance to 4-HPR [28]. Protein expression analysis showed that A2780 cells constitutively expressed AF1q protein and that treatment with 5 and 10 µM 4-HPR for 24 hours increased its expression in a dose-dependent manner (Figure 1A). In contrast, 4-HPR treatment did not cause any increase in AF1q protein expression in A2780/HPR cells, even though the cells constitutively expressed it (Figure 1B). Such data suggested an association between AF1q induction by 4-HPR treatment and cell sensitivity to the retinoid.

### 4-HPR Enhances AF1q Expression in Other Ovarian Cancer Cells and in Cancer Cell Lines of Different Histotypes

**Figure 2:**
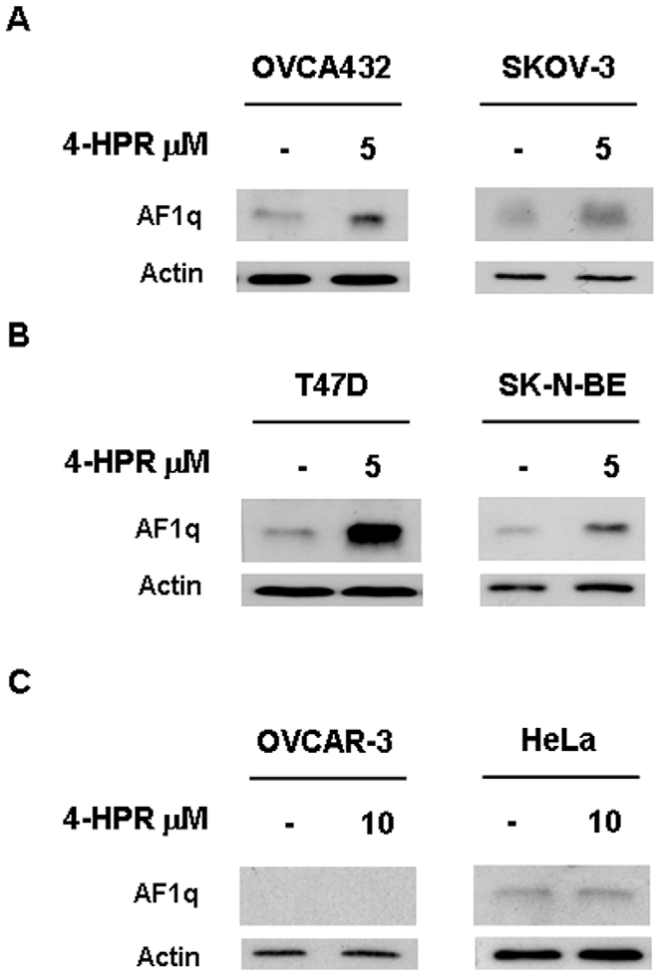
Effects of 4-HPR treatment on AF1q expression in a panel of cancer cell lines. Western blot analysis for AF1q expression in OVCA432 and SKOV-3 (A) cells, in T47D and SK-N-BE (B) cells, and in OVCAR-3 and HeLa (C) cells treated for 24 hours with 4-HPR at the indicated doses. As a control for loading, the blots were incubated with actin antibody.

To assess whether 4-HPR-induced AF1q upregulation was restricted to A2780 cells or represented a distinctive mode of action of the retinoid, we extended the analysis of AF1q expression to others human cancer cells with different sensitivities to the antiproliferative effect of 4-HPR. Treatment with 5 µM 4-HPR for 24 hours induced AF1q upregulation in the sensitive ovarian cancer cell lines OVCA432 and SKOV-3 [27] (Figure 2A), as well as in responsive cells of different histotypes, i.e., mammary adenocarcinoma T47D and neuroblastoma SK-N-BE [27] (Figure 2B). In contrast, in cells naturally resistant to 4-HPR growth inhibitory activity such as the ovarian cancer cell line OVCAR-3 [27] and the cervical carcinoma cell lines HeLa (IC_50_>10 µM, at 72 hours, data not shown), neither 5 µM (data not shown) nor 10 µM 4-HPR treatment for 24 hours caused upmodulation of AF1q expression (Figure 2C). The results suggested that 4-HPR-induced AF1q upregulation did not occur only in A2780 cells but was a characteristic of cancer cells responsive to the retinoid.

### AF1q Upregulation in A2780 Cells is Associated to Retinoid Ability to Inhibit Cell Growth and Induce Apoptosis

**Figure 3:**
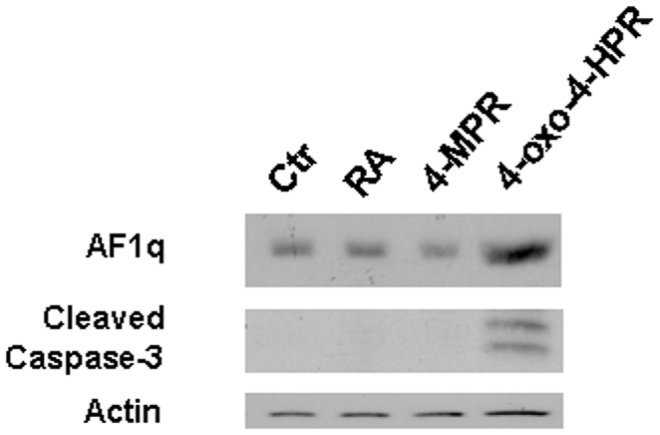
Effects of different retinoids on AF1q expression in A2780 cells. Western blot analysiso for AF1q expression and caspase-3 cleavage in A2780 cells treated for 24 hours with 10 µM RA or 4-MPR, or 3 µM 4-oxo-4-HPR. As a control for loading, the blot was incubated with actin antibody.

Analysis of the association between AF1q expression and tumor growth inhibitory and apoptotic effects was extended to other natural and synthetic retinoids, i.e., RA, and N-(4-methoxyphenyl)retinamide (4-MPR) and 4-oxo-N-(4-hydroxyphenyl)retinamide (4-oxo-4-HPR), two 4-HPR metabolites. We previously demonstrated that 4-oxo-4-HPR has a growth inhibitory effect (IC_50_ = 0.6 µM, at 72 hours) and induces apoptosis in A2780 cells, whereas 4-MPR and RA do not affect such growth (IC_50_>10 µM, at 72 hours for both retinoids) [27]–[29]. As shown in Figure 3, in A2780 cells the treatment with RA, as well as with 4-MPR, did not affect either AF1q expression or caspase-3 activation, whereas 4-oxo-4-HPR treatment led to an increase in AF1q expression and to caspase-3 cleavage (Figure 3). The data demonstrated that retinoid-induced AF1q upregulation was associated with the ability of the compound to inhibit cell growth and to induce apoptosis.

### AF1q Upregulation is Functionally Involved in Apoptosis Induced by 4-HPR in A2780 Cells

**Figure 4:**
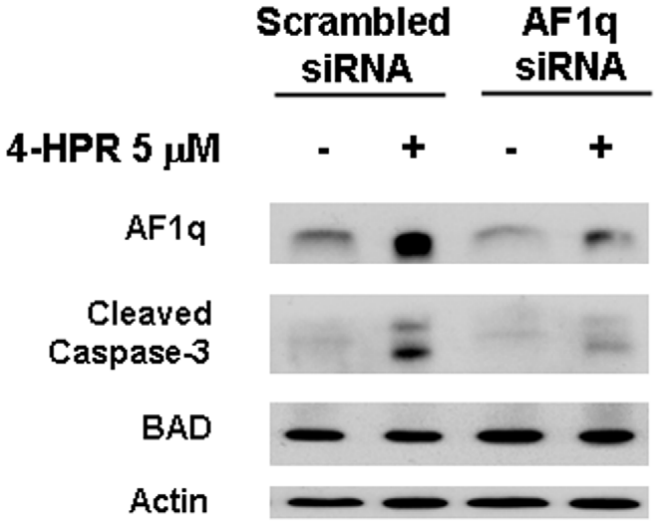
Role of AF1q upmodulation in 4-HPR-induced apoptosis. Western blot analysiso for AF1q expression, caspase-3 cleavage, and BAD expression in A2780 cells transiently transfected with a plasmid containing a AF1q siRNA or a scrambled nonsilencing siRNA following addition of 5 µM 4-HPR for 24 hours. As a control for loading, the blot was incubated with actin antibody.

To study the potential role of AF1q in 4-HPR-mediated apoptosis, we performed AF1q silencing by transient transfections of A2780 cells with a plasmid expressing an AF1q siRNA or a scrambled nonsilencing siRNA used as control. Transfection with AF1q siRNA reduced both basal and 4-HPR-induced AF1q expression and clearly decreased 4-HPR-induced apoptosis, as shown by cleavage of caspase-3 (Figure 4), demonstrating that AF1q played a role in apoptosis induction by 4-HPR in A2780 cells. Under the same experimental conditions, we did not observe modulation of the proapoptotic protein BAD (Figure 4), previously shown to be regulated by AF1q in human squamous carcinoma cells [24], [25].

### AF1q is Involved in the Apoptotic Signaling Cascade Induced by 4-HPR in A2780 Cells

**Figure 5:**
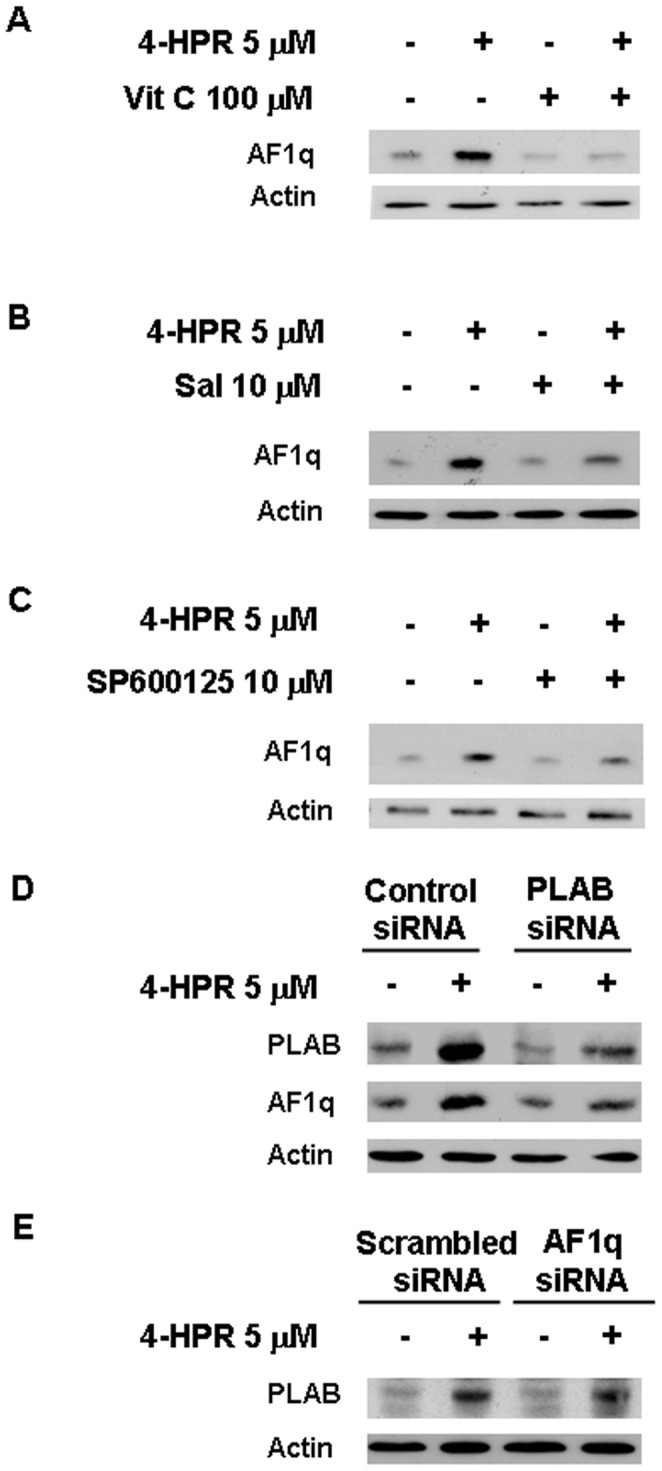
Relationship between AF1q upregulation and 4-HPR-induced signaling cascade. Western blot analysis for AF1q expression in A2780 cells treated for 24 hours with 5 µM 4-HPR, with or without 100 µM vitamin C (A), 10 µM salubrinal (B), or 10 µM SP600125 (C). (D) Western blot analysis for AF1q expression in A2780 cells stably transfected with a plasmid containing a PLAB siRNA or a scrambled nonsilencing siRNA following addition of 5 µM 4-HPR for 24 hours. As a control for loading, the blots were incubated with actin antibody.

**Figure 6:**
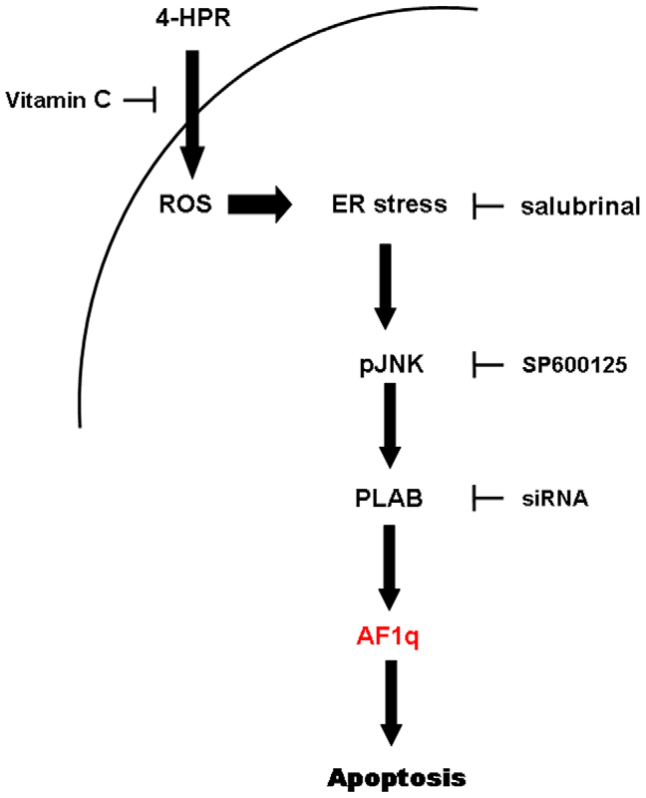
Scheme showing proposed cascade of events involved in 4-HPR-induced growth inhibitory effect. 4-HPR induces apoptosis through a signaling cascade involving oxidative stress, ER stress response, JNK activation, overexpression of the proapoptotic protein PLAB, and AF1q upregulation.

We previously reported that 4-HPR-induced apoptosis occurs through a signaling cascade involving ROS generation, ER stress induction, JNK activation and PLAB upregulation (ROS → ER stress → JNK → PLAB) [11], [12]. We thus evaluated whether AF1q was involved in the aforementioned apoptotic pathway by testing the effects of the inhibition of these signaling intermediates on 4-HPR-induced AF1q upmodulation. To this aim, A2780 cells were co-treated with 4-HPR and, in turn, with the antioxidant vitamin C, the ER stress inhibitor salubrinal, and the pharmacological JNK inhibitor SP600125, which have been demonstrated to prevent ROS generation, eIF2α dephosphorylation, and JNK activation, respectively, and to strongly reduce 4-HPR-induced apoptosis in the same cell line [11]. The addition of 100 µM vitamin C to A2780 cells treated with 5 µM 4-HPR for 24 hours abrogated the 4-HPR-induced upregulation of AF1q (Figure 5A). Similarly, the addition of 10 µM salubrinal or 10 µM SP600125 strongly reduced 4-HPR-induced AF1q upregulation (Figure 5B and 5C). To investigate whether the upregulation of AF1q was a downstream event also of PLAB upregulation induced by 4-HPR, PLAB has been knocked down using a synthetic siRNA targeting PLAB mRNA. As shown in Figure 5D, the treatment of A2780 cells with the PLAB-specific siRNA was able to strongly reduce 4-HPR-induced PLAB as well as AF1q upmodulation compared with the cells transfected with a control siRNA. Conversely, AF1q silencing did not affect 4-HPR-induced PLAB upregulation (Figure 5E). The aforementioned data indicated that in A2780 cells AF1q upregulation was dependent on prior activation of the 4-HPR-induced-ROS-related signaling cascade and that its upmodulation occurred downstream of PLAB induction (Figure 6).

### AF1q Plays a Role in the Induction of Basal Apoptosis in Ovarian Cancer Cells

**Figure 7:**
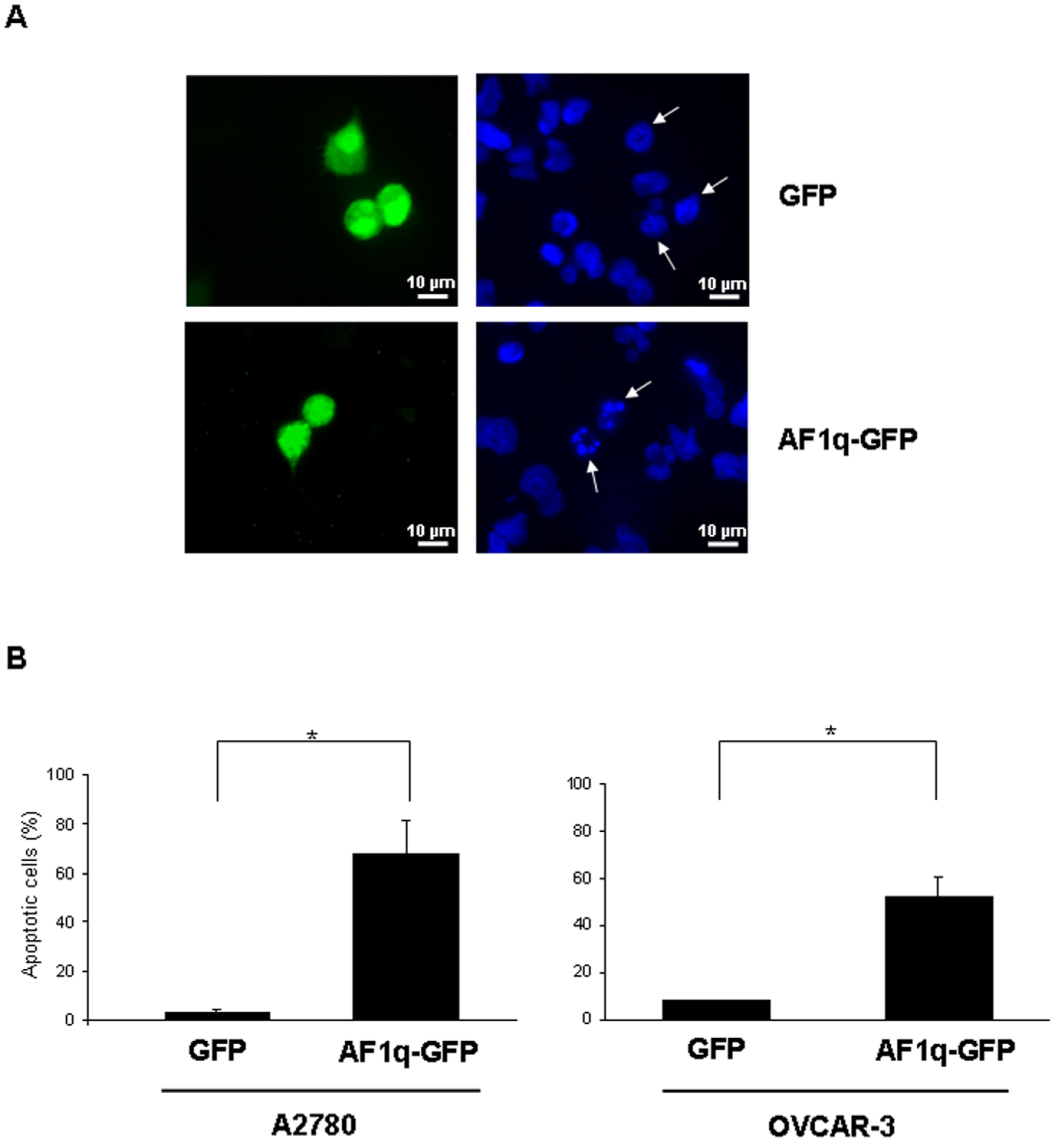
Effect of AF1q overexpression in the onset of basal apoptosis. (A) Immunofluorescence analysis of A2780 cells transiently transfected with GFP alone (upper panels) or GFP-tagged AF1q vector (AF1q-GFP) (lower panels). After 48 hours, GFP and AF1q-GFP cells were stained with Hoechst 33342 and nuclear morphology was examined with a fluorescent microscope. Cells of interest are marked by arrows. Cells with condensed and fragmented nuclei were identified and scored as apoptotic cells. One experiment representative of three is shown. The scale bar represents 10 µm. (B) Apoptosis was represented as percentage of apoptotic cells per 100 green fluorescent cells (at least 200 cells per sample) in A2780 (left panel) and OVCAR-3 (right panel) cells transfected as in (A). Data represent the mean±S.D. of three independent experiments. Asterisks indicate significant difference (P<0.05).

To investigate the link between AF1q induction and apoptosis onset, we analyzed whether direct overexpression of AF1q, in the absence of external stimuli, could cause apoptosis. To this aim, A2780 cells were transiently transfected with a Green Fluorescent Protein (GFP)-tagged AF1q vector. Cells transfected with a vector coding for GFP were used as control. At 48 hours after transfection, nuclear fragmentation and chromatin condensation were monitored in green fluorescent cells by staining cell nuclei with Hoechst 33342 (Figure 7A). As shown in Figure 7B (left panel), GFP-AF1q-overexpressing cells showed a marked increase in apoptotic nuclei relative to control cells transfected with GFP alone. A similar effect was observed when the analysis was performed in OVCAR-3 cells, another ovarian cancer cell line that, differently from A2780 cells, was resistant to the 4-HPR antiproliferative effect and did not constitutively express AF1q. Transfection with GFP-AF1q induced also in OVCAR-3 an increase in basal apoptosis relative to transfection with GFP (Figure 7B, right panel). These findings suggested a role for AF1q in the onset of apoptosis in ovarian cancer cells.

## Discussion

The recent findings that the AF1q gene, beside its documented oncogenic function [17]–[23], plays a role in apoptosis regulation in cancer cells [24], [25] and the fact that it has been described as an RA-target gene [14], prompted us to investigate its possible involvement in apoptosis induced by the synthetic nonclassic retinoid 4-HPR. The present study identified AF1q as a novel mediator of apoptosis induced by 4-HPR and provided evidence of a role of the protein in the onset of basal apoptosis in ovarian cancer cells. We showed that in A2780, a human ovarian carcinoma cell line highly sensitive to the growth inhibitory and apoptotic effects of the retinoid [26], [27], 4-HPR-induced apoptosis was accompanied by an increase in AF1q protein expression. In contrast, the upmodulation of AF1q was not observed when A2780/HPR cells, a 4-HPR resistant variant derived from A2780 cells [28], were treated with the retinoid. Analysis of AF1q expression, extended to a panel of human cancer cell lines (ovarian, breast, cervical, and neuroblastoma) with different sensitivities to the 4-HPR antiproliferative effect [27], confirmed that upmodulation of the protein after 4-HPR exposure was a common feature of 4-HPR responsive cells but did not occur in cancer cells resistant to the retinoid.

The association between cell sensitivity to 4-HPR anticancer effects and AF1q upregulation suggested a role for AF1q as a putative mediator of 4-HPR activity. The hypothesis was also supported by the fact that AF1q expression was selectively upregulated by 4-HPR and by its active metabolite 4-oxo-4-HPR, but not by RA or 4-MPR, which failed to induce cell death in A2780 cells [27]–[29]. The lack of induction of AF1q and apoptosis after challenging the cells with RA is consistent with the issue that 4-HPR biological effects and the mechanisms underlying its anticancer activities greatly differ from those of classical retinoids [9] and are instead more close to those described for atypical retinoids [30]. A direct link between AF1q upregulation and apoptosis induced by 4-HPR was demonstrated by AF1q silencing, which led to a reduction in 4-HPR-induced apoptosis, thus indicating that AF1q is, at least in part, responsible for the apoptotic effects caused by the retinoid in ovarian cancer cells.

We previously showed that 4-HPR was able to cause apoptosis in ovarian cancer cells through the activation of the ROS → ER stress → JNK → PLAB signaling cascade [11], [12]. Induction of such a cascade was characteristic only of cancer cells responsive to 4-HPR and was not activated in 4-HPR-resistant cells [12]. We thus hypothesized that 4-HPR-induced upregulation of AF1q, which occurred only in cells sensitive to the retinoid, could be an intermediate of the aforementioned apoptotic pathway. Consistent with this hypothesis, we found that the upregulation of AF1q occurred downstream of ROS generation, ER stress response, JNK activation, and PLAB upregulation induced by 4-HPR. In fact, by inhibiting the pathway at different levels using in turn the antioxidant vitamin C, the selective inhibitor of eIF2α dephosphorylation salubrinal, and the JNK inhibitor SP600125 (all previously proved to protect A2780 cells from 4-HPR-induced apoptosis [12]), we showed that 4-HPR-induced AF1q upregulation was strongly reduced. Moreover, silencing of the proapoptotic protein PLAB (previously shown to reduce 4-HPR-induced apoptosis [11]) caused a decreased ability of the retinoid to increase AF1q expression. Conversely, AF1q silencing did not affect the ability of 4-HPR to induce PLAB upregulation The aforementioned results demonstrated that AF1q was an intermediate of the ROS-related signaling cascade activated by 4-HPR to induce apoptosis and that AF1q upmodulation occurred downstream of PLAB induction (Figure 6). However, the contribution of other ROS-dependent signals to the 4-HPR-induced AF1q upmodulation cannot be excluded. Not surprisingly, we found that AF1q was upregulated also after treatment with 4-oxo-4-HPR, a 4-HPR polar metabolite able to activate the aforementioned apoptotic cascade (from ROS generation to PLAB induction) [31].

In agreement with our observations, analysis of one publicly available microarray experiment dataset on 4-HPR-treated and untreated CD34+ cells from four chronic myeloid leukemia patients (GEO Accession: GSE17480) showed that the retinoid induced a six-fold increase in AF1q mRNA expression (P<0.01) (Figure S1). In the same cells, 4-HPR was able to induce apoptosis - suggested to be mediated by the induction of oxidative stress, thus confirming the relationship between ROS mediated-apoptosis induced by 4-HPR and AF1q overexpression.

According to the apoptotic role suggested by our observations, AF1q involvement in apoptosis regulation and in drug resistance has been reported by another research group [24], [25]. The authors showed that in human squamous carcinoma cells, AF1q downregulation was associated with a doxorubicine-resistant cell phenotype and that AF1q upmodulation caused an increased doxorubicin and γ radiation-induced apoptosis through transactivation of the proapoptotic protein BAD via NF-kB [24], [25]. In contrast, we did not observe upregulation of BAD in AF1q-mediated apoptosis induced by 4-HPR in A2780 cells. In fact, 4-HPR treatment did not modulate BAD expression, nor did AF1q silencing cause changes in its protein levels. One possible explanation could be that AF1q might differently affect BAD expression in different cancer cell types and/or in response only to specific stimuli. However, it is interesting to note that, as for 4-HPR and 4-oxo-4-HPR [12], [31], doxorubicin and γ radiation-induced apoptosis has also been reported to be associated to ROS generation [32]–[34]. Therefore, it is tempting to speculate that AF1q-mediated apoptosis could depend on prior induction of oxidative stress, not only for 4-HPR and 4-oxo-4-HPR but also for other chemotherapeutic agents, although at present no sufficient evidence supports such a hypothesis.

An important finding of our study is the indication of a role of AF1q in the onset of basal apoptosis: AF1q upmodulation obtained by transient transfection in ovarian cancer cells caused, in the absence of external stimuli, an increase in the apoptotic rate, thus demonstrating a role of AF1q in actively inducing apoptosis in these cells. A comprehensive understanding of the precise physiological and pathological functions of AF1q is definitely in its infancy. However, the few studies that have investigated its role revealed that it might be implicated in both inhibition and promotion of cancer progression [17]–[25]. In addition to evidence of a role as an apoptosis mediator, AF1q has been defined as an oncogenic factor involved in hematological malignancies as well as in solid tumors [17]–[23]. It is noteworthy to observe that, similarly to that reported for AF1q, PLAB has also been shown to be endowed with a dual function in cancer progression [35]. In fact, this member of the transforming growth factor-β superfamily can in fact negatively or positively modulate cell proliferation and apoptosis and may contribute to cancer migration, invasion, and metastasis. Even though the mechanisms regulating the pleiotropic functions of PLAB are still not well understood, it has been proposed that the protein can act as a tumor suppressor in the early stages of malignancy, whereas it can promote tumor progression in advanced stages of cancer [35]. An intriguing consideration is that PLAB and AF1q have other features in common: they are both RA-target genes and are able to induce apoptosis in the absence of external stimuli [11], [36]. Although further studies are necessary to define the exact functions of AF1q and to investigate the possible association between PLAB and AF1q, the present data demonstrate for the first time a relationship between these two proteins.

In conclusion, our study expands the knowledge of the mechanisms of action of 4-HPR that, even though investigated for many years, have not yet been completely elucidated [8]. The identification of novel targets of the retinoid is an important issue because it might lead to facilitate future design of drug combination strategies and to overcome and prevent the development of drug resistance. In addition, we provide new information about the activity of AF1q, a cancer-related protein whose biological functions are largely unknown. Specifically, we report that AF1q is involved in 4-HPR apoptotic and growth inhibitory effects and that its upregulation depends on the activation of the ROS-related signaling cascade induced by the retinoid, thus discovering a novel relationship between oxidative stress and AF1q. Finally, we demonstrate for the first time a role for AF1q in the induction of basal apoptosis in ovarian cancer cells, although additional studies are needed to define the molecular mechanisms underlying AF1q-mediated apoptosis.

## Materials and Methods

### Cell Lines and Reagents

The ovarian tumor cell lines A2780 (obtained from Dr. Ozols, Bethesda, MD, USA) [31], OVCA432 (obtained from Dr. Knapp, Boston, MA, USA) [12], OVCAR-3 [12], SKOV-3 [12], and the neuroblastoma cell line SK-N-BE (all three purchased from ATCC, Manassas, VA, USA) [31] were maintained in RPMI 1640 (Lonza, Basel, Switzerland) containing 10% fetal calf serum. A2780/HPR (i.e., A2780 cells made resistant to 4-HPR as previously described [28]) cells were maintained in RPMI 1640 containing 10% fetal calf serum supplemented with 5 µM 4-HPR. The breast tumor cell line T47D (obtained from Dr. R. Sutherland, Sydney, New South Wales, Australia) [31] was maintained in RPMI 1640 containing 10% fetal calf serum and 0.25 U/ml insulin. The cervical carcinoma cell line HeLa, purchased from ATCC [31], was maintained in Dulbecco’s Modified Eagle medium (Gibco Brl, Paisley, UK) supplemented with 10% fetal calf serum. All cell lines were cultured at 37°C under 5% CO2. 4-HPR, 4-MPR (both kindly provided by Dr. J. Crowell, National Cancer Institute, Bethesda, MD, USA), RA (purchased from Sigma, St Louis, MO, USA), and 4-oxo-4-HPR (synthesized as previously described [29]) were dissolved at 10 mM in DMSO, prior to dilution in culture medium and stored at –80°C in the dark. Vitamin C (Sigma) and JNK inhibitor SP600125 (Calbiochem, San Diego, CA, USA) were added to cells 60 minutes and 15 minutes before 4-HPR, respectively. ER stress response inhibitor salubrinal (Calbiochem) was added together with 4-HPR.

### Immunoblot Analysis

Proteins were extracted by lysing cells in sodium dodecyl sulfate (SDS) sample buffer (62.5 mM Tris–HCl [pH 6.8], 2% SDS) containing 1 mM phenylmethylsulfonyl fluoride, 10 µg/mL pepstatin, 12.5 µg/mL leupeptin, 2 µg/mL aprotinin, 1 mM sodium orthovanadate, and 1 mM sodium molybdate. Cell extracts were processed for western immunoblotting as previously described [37]. The following antibodies used for immunoblotting were purchased from the indicated suppliers: AF1q from Abnova (Taipei City, Taiwan, #H00010962-M01); cleaved Caspase-3 (Asp175) from Cell Signaling Biotechnology (Beverly, MA, USA, #9661); BAD from Transduction Laboratories (Lexington, KY, USA, #610391); PLAB from R&D Systems (Minneapolis, MN, USA, #BAF940); and actin from Sigma (#A2066).

### Immunofluorescence Analysis

Cells, grown and transfected on glass coverslip slides in 24 mm Petri dishes, were fixed in 4% paraformaldehyde at room temperature for 10 minutes, washed with PBS and then stained with Hoechst 33342 (Sigma) 2 µg/ml in PBS for 2 minutes. Slides were mounted with 0.1% (v/v) Mowiol (Calbiochem) and viewed with a fluorescence microscope [images were recorded with a Spot Insight digital camera (Delta Sistemi) equipped with a system of image analysis (IAS 2000; Delta Sistemi)]. Apoptotic cells were identified by analysis of nuclear morphology. Green florescent cells with condensed and fragmented nuclei were identified and scored as apoptotic cells (at least 200 cells per sample).

### Construction of GFP-tagged AF1q Expression Vector

AF1q was transiently expressed as a fusion protein with the GFP fused to its C-terminal. AF1q coding the cDNA region deprived of the stop codon was obtained by Polymerase Chain Reaction (PCR) amplification from a plasmid containing the cDNA encoding human AF1q obtained from the Integrated Molecular Analysis of Genomes and their Expression (I.M.A.G.E.) Consortium (Livermore, CA, USA) (cDNA clone IMAGE:305990). We used the forward oligonucleotide primer 5′-ACTCGAGCCACCATGAGGGACCCTGTGAG-3′ [which contains an XhoI restriction site and a Kozak sequence (CCACC)], and the reverse oligonucleotide primer 5′-ATCCGGATCCAGCAAGTCCAGTTCG -3′ (which contains a BamHI restriction site). The amplification reaction was initiated by incubation of the sample at 94°C for 2 minutes, followed by 15 cycles consisting of incubation at 94°C for 30 s, at 45°C for 60 s and 68°C for 90 s and by 20 cycles at 94°C for 60 s and 68°C for 90 s. The resultant PCR fragment was digested with XhoI and Bam HI and ligated in frame with the GFP gene into the expression vector pEGFP-N1 (Clontech, Palo Alto, CA, USA), previously digested with the same enzymes.

### AF1q Knockdown

Cells (5×10^5^) were seeded in 60 mm dishes; 24 hours later, a mixture of Lipofectamine 2000 reagent (Invitrogen, San Diego, CA, USA) and 8 µg of expression plasmid was added and then incubated for 6 hours. Cells were then cultured in medium supplemented with 10% serum for an additional 48 hours before further analysis. Plasmids expressing a AF1q siRNA and a scrambled nonsilencing siRNA were purchased from Origene (Rockville, MD, USA, #TF319110).

### PLAB Knockdown

A2780 cells were transfected with a synthetic PLAB siRNA and with a All Stars Negative Control siRNA (from the Flexitube Gene Solution siRNA kit of Qiagen, Milan, Italy, #GS9518 and #SI03650318). Transfections were performed using 32 nM siRNA in OptiMEM medium (Invitrogen) with Lipofectamine 2000 (1%, v/v), following the manufacturer’s instruction. Forty-eight hours post-transfection, the cells were treated or not with 5 µM 4-HPR for 24 hours and PLAB knockdown was confirmed by Western blot analysis.

### Statistical Analysis

Experiments were carried out at least in triplicate. Differences between mean values were assessed by Student’s t-test with two-sided P values <0.05 considered as statistically significant.

## Supporting Information

Figure S1 **AF1q expression in CD34+ cells from CML patients with or without 4-HPR treatment.** AF1q expression levels were extracted from the GSE17480 dataset, where the expression profile of CD34+ cells from CML patients after treatment or not with 4-HPR was reported. Using a paired t-test, AF1q expression was found to be significantly higher in 4-HPR treated cells.
(TIF)
