# Supplementary figures and images for: AF1q: A Novel Mediator of Basal and 4-HPR-Induced Apoptosis in Ovarian Cancer Cells
Source: PLoS One. 2012 Jun 26;7(6):e39968. doi: 10.1371/journal.pone.0039968 (PMC3383705; doi:10.1371/journal.pone.0039968)

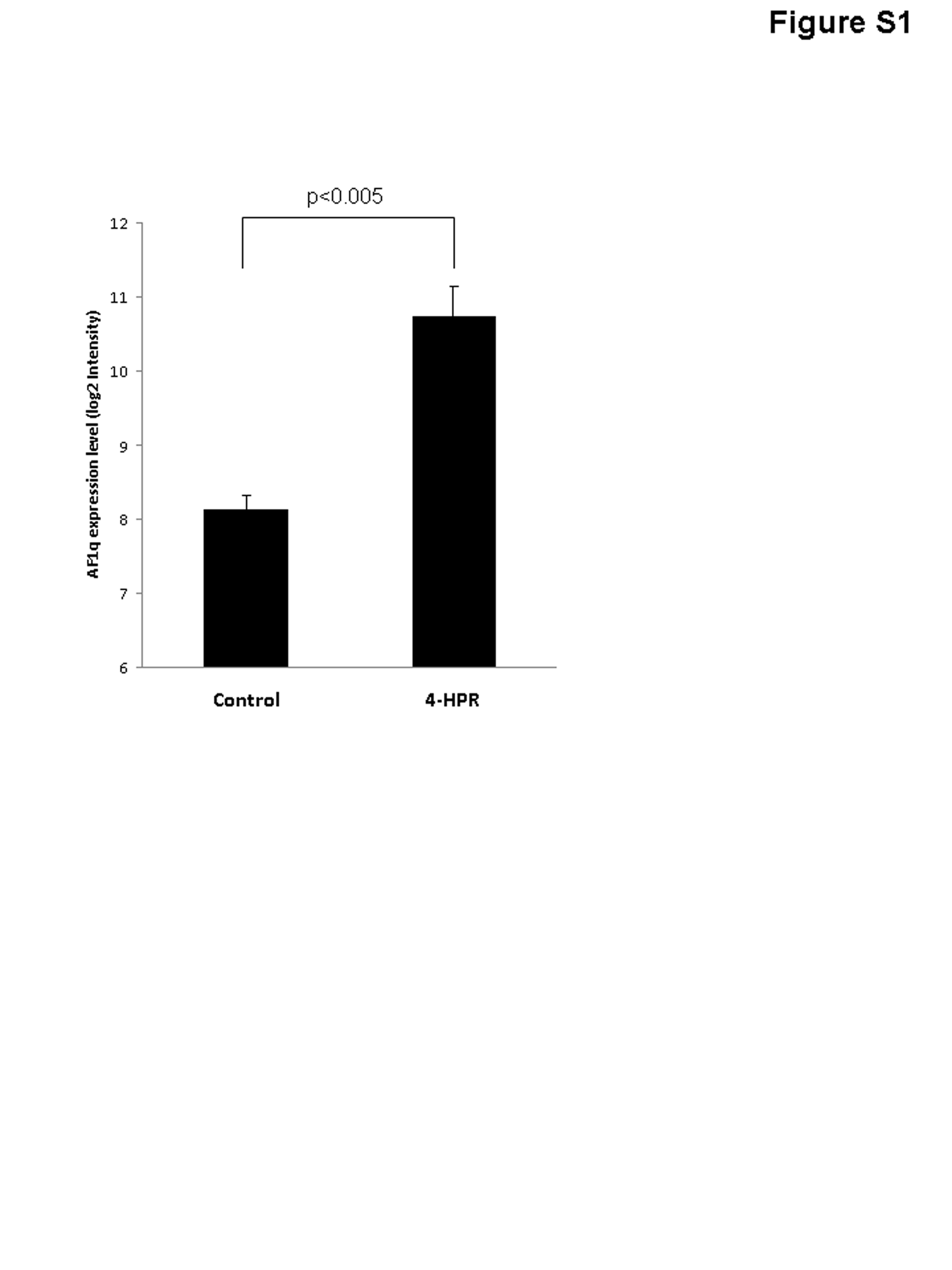

Supplement: Figure S1 — AF1q expression in CD34+ cells from CML patients with or without 4-HPR treatment. AF1q expression levels were extracted from the GSE17480 dataset, where the expression profile of CD34+ cells from CML patients after treatment or not with 4-HPR was reported. Using a paired t-test, AF1q expression was found to be significantly higher in 4-HPR treated cells. (TIF) [file pone.0039968.s001.tif]
